# Brainwide mesoscale functional networks revealed by focal infrared neural stimulation of the amygdala

**DOI:** 10.1093/nsr/nwae473

**Published:** 2024-12-24

**Authors:** An Ping, Jianbao Wang, Miguel Ángel García-Cabezas, Lihui Li, Jianmin Zhang, Katalin M Gothard, Junming Zhu, Anna Wang Roe

**Affiliations:** Department of Neurosurgery of the Second Affiliated Hospital and Liangzhu Laboratory, Zhejiang University School of Medicine, Hangzhou 310009, China; Interdisciplinary Institute of Neuroscience and Technology, School of Medicine, Zhejiang University, Hangzhou 310029, China; School of Medicine, Zhejiang University, Hangzhou 310058, China; Department of Neurosurgery of the Second Affiliated Hospital and Liangzhu Laboratory, Zhejiang University School of Medicine, Hangzhou 310009, China; Interdisciplinary Institute of Neuroscience and Technology, School of Medicine, Zhejiang University, Hangzhou 310029, China; School of Medicine, Zhejiang University, Hangzhou 310058, China; MOE Frontier Science Center for Brain Science and Brain-machine Integration, Zhejiang University, Hangzhou 310012, China; Department of Anatomy, Histology, and Neuroscience, School of Medicine, Autónoma University of Madrid, Madrid 28049, Spain; Interdisciplinary Institute of Neuroscience and Technology, School of Medicine, Zhejiang University, Hangzhou 310029, China; Key Laboratory for Biomedical Engineering of Ministry of Education, College of Biomedical Engineering and Instrument Science, Zhejiang University, Hangzhou 310027, China; Department of Neurosurgery of the Second Affiliated Hospital and Liangzhu Laboratory, Zhejiang University School of Medicine, Hangzhou 310009, China; Departments of Physiology and Neuroscience, University of Arizona, Tucson 85721, USA; Department of Neurosurgery of the Second Affiliated Hospital and Liangzhu Laboratory, Zhejiang University School of Medicine, Hangzhou 310009, China; Department of Neurosurgery of the Second Affiliated Hospital and Liangzhu Laboratory, Zhejiang University School of Medicine, Hangzhou 310009, China; Interdisciplinary Institute of Neuroscience and Technology, School of Medicine, Zhejiang University, Hangzhou 310029, China; School of Medicine, Zhejiang University, Hangzhou 310058, China; MOE Frontier Science Center for Brain Science and Brain-machine Integration, Zhejiang University, Hangzhou 310012, China; Key Laboratory for Biomedical Engineering of Ministry of Education, College of Biomedical Engineering and Instrument Science, Zhejiang University, Hangzhou 310027, China

**Keywords:** infrared neural stimulation, amygdala, ultrahigh field fMRI, brainwide network, mesoscale topography, connectional topography, intra-individual datasets

## Abstract

The primate amygdala serves to evaluate the emotional content of sensory inputs and modulate emotional and social behaviors; it modulates cognitive, multisensory and autonomic circuits predominantly via the basal, lateral and central nuclei, respectively. Recent evidence has suggested the mesoscale (millimeter-scale) nature of intra-amygdala functional organization. However, the connectivity patterns by which these mesoscale regions interact with brainwide networks remain unclear. Using infrared neural stimulation of single mesoscale sites coupled with mapping in ultrahigh field 7-T functional magnetic resonance imaging, we have discovered that these mesoscale sites exert influence over a surprisingly extensive scope of the brain. Our findings strongly indicate that mesoscale sites within the amygdala modulate brainwide networks through a ‘one-to-many’ (integral) way. Meanwhile, these connections exhibit a point-to-point (focal) topography. Our work provides new insights into the functional architecture underlying emotional and social behavioral networks, thereby opening up possibilities for individualized modulation of psychological disorders.

## INTRODUCTION

The primate amygdala is an important center that evaluates the emotional salience of inputs from all sensory modalities and contributes to the elaboration of emotional and social behaviors [1,2]. Anatomical, electrophysiological and behavioral studies indicate that the integration and dissemination of neural information across sensory-motor and decision networks is achieved by multiple functional processing loops connecting the amygdala to a wide array of cortical and subcortical targets [3–5]. Anatomical studies have shown that three major subdivisions of the amygdala project to distinct targets in the brain, suggesting functional subdivisions for extending the amygdala's influence across the brain. Specifically, the central subnucleus (CeA) projects primarily to targets in the basal forebrain, hypothalamus and brainstem [6], the basal subnucleus (BA) projects to large swaths of the frontal, insular, temporal and visual cortex, as well as claustrum and cingulate cortex [7,8], and the lateral subnucleus (LA) gives rise to projections to orbitofrontal, cingulate and insular cortex, as well as hippocampal areas [9]. Thus, functional distinctions in connectivity associate CeA, BA and LA broadly with autonomic, cognitive and multisensory circuits, respectively [10,11].

**Figure 1.**
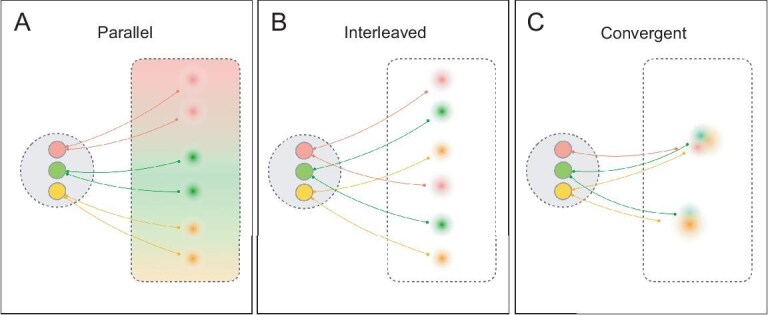
Possible mesoscale connectivity patterns. Colored circles: mesoscale domains. Areas surrounded by dashed lines: brain areas.

At question is how the amygdala mediate its influence with existing functional organizations in target cortical areas. There is a broad recognition that the fundamental organization of the human brain is based in mesoscale (millimeter-scale) information units (columns, domains). Much of the evidence comes from studies in non-human primates in which (i) moving an electrode by ∼500 um leads to different response preferences, revealing submillimeter clusters of functionally specific cells; (ii) optical imaging and functional magnetic resonance imaging (fMRI) studies of such cellular clusters reveal populations of functionally distinct mesoscale (millimeter-scale) domains; and (iii) injections of small volumes of anatomical tracers into single functional domains reveal highly specific intra-areal and inter-areal mesoscale local networks. Recent studies have discovered that these clusters are synaptically connected to form structurally parallel (e.g. color-related and shape-related) mesoscale circuits within and between areas in the primate visual cortex [12]. Such mesoscale organizational principles have been well established in the visual system but extend to other sensory, motor, parietal and prefrontal areas as well (as evidenced by anatomical [13], optical imaging [14], and high-resolution fMRI studies [15,16]) (Fig. 1).

Within the subcortical amygdala, submillimeter clustering is also seen. Anatomical studies have suggested the presence of further subdivisions within subnuclei of the amygdala (e.g. magnocellular (dorsal) vs parvicellular (ventral) BA associated with occipital vs anterior visual areas [8,17]). Perhaps the strongest evidence for internal clustering comes from local field potential and current source density recordings in macaque amygdala, which have revealed a distinct (e.g. visual, tactile, auditory and multisensory) mesoscale functional organization within the amygdala in behaving monkeys [18,19], the integration of which is central to the interpretation of social facial communications [18]. These data [18,19] raise the exciting possibility that connectional relationships of the amygdala with other brain areas are also organized at mesoscale. Here, we hypothesize that amygdalar influences interface with functionally selective mesoscale nodes in target cortical areas.

There are multiple methods for studying brainwide circuits. Anatomical studies typically reveal only direct connections with other brain areas; polysynaptic connections can be revealed (e.g. with rabies viruses), but such studies in macaques are few [20,21]. Functional resting state data provide brainwide information [10,22], but generally do not have the resolution to address connection patterns at mesoscale. Recently, we developed a method to address this need, termed infrared neural stimulation in ultrahigh field fMRI (INS-fMRI). Brief pulses (0.25 msec) of 1875 nm wavelength light are delivered via 200 um diameter fiber optics to stimulate submillimeter sites in the brain; these tiny heat boli lead to membrane capacitance changes and neuronal firing [23]. The activation is focal (within ∼500 um) and non-damaging [24,25], as illustrated by histology, electrophysiology, optical imaging, fMRI, behavior, MRI thermometry [26] and modeling studies of temperature increase [27] (for review of INS, see [28–30]). Unlike optogenetics, this method is non-viral and can be used to stimulate any site in the brain without prior preparation. Functional activations of connected sites are mapped at full brain scale by recording blood oxygen-level dependent (BOLD) signals. Importantly, as INS reveals mono- and di-synaptic functional connectivity (1 site: 1 functional network) [31], it evokes a broader, yet highly specific, set of network activations, beyond what traditional anatomical tracing provides [32,33]. No animal sacrifice is needed, permitting data acquisition from multiple sites of stimulation within a single animal; this provides a rich dataset of multiple mesoscale networks whose organization and mutual relationships can be compared. INS thus provides distinct uses for high resolution circuit mapping [34,35] in primate brains.

Using this method, we previously established proof-of-principle that INS-fMRI in macaque amygdala BA reveals statistically significant, mesoscale connectivity with connected sites in the cingulate, insular and association sensory cortex [32,33]. Here, we provide a more extensive study of brainwide targets of LA, BA and CeA in single individual macaque monkeys. We show that amygdalar networks are composed of mesoscale nodes, are highly functionally specific, and form an elegant functional architecture for mediating emotion-related influences across multiple cortical areas. This new understanding of brain function will impact how clinicians interface with the brain in disease, how engineers design brain interface technologies, and how computational neuroscientists model functional brainwide networks.

## RESULTS

### Overview

The purpose of this study was to examine the organization of connections between the amygdala and various cortical areas in individual macaque monkeys. We note that, compared with most anatomical studies where tracer injections can span several millimeters, our stimulation is significantly more focal, activating, with intensities used, a volume of tissue <1 mm^3^ [36]. A major strength of the INS method is that networks activated by the stimulation of multiple sites can be compared within an individual. A weakness is that, due to the focal nature of the stimulation, we are sampling a small volume of the total amygdala. This study presents a sampling of nuclei CeA, BA and LA and reveals the mesoscale aspect of their connection patterns. Our rationale for analysis of the data begins with a matrix-based comparison of known anatomy and the presence or absence of INS-evoked connections. Further, as functional connections include both ‘first synapse’ and ‘second synapse’ connections, the functional connectivity networks are expected to be greater than the anatomical connectivity networks. Note that, because INS-fMRI is biased in the ‘anterograde’ direction, these connections reflect more strongly amygdalofugal functional connectivity. We then examine, within each of the cortical areas (which span limbic, sensory, motor, cognitive and prefrontal cortical areas), the spatial distribution of connections. The examples shown highlight connection patterns in limbic cortical areas as well as topographic and interleaved connection patterns.

### INS of the amygdala reveals remote connections at mesoscale

**Figure 2.**
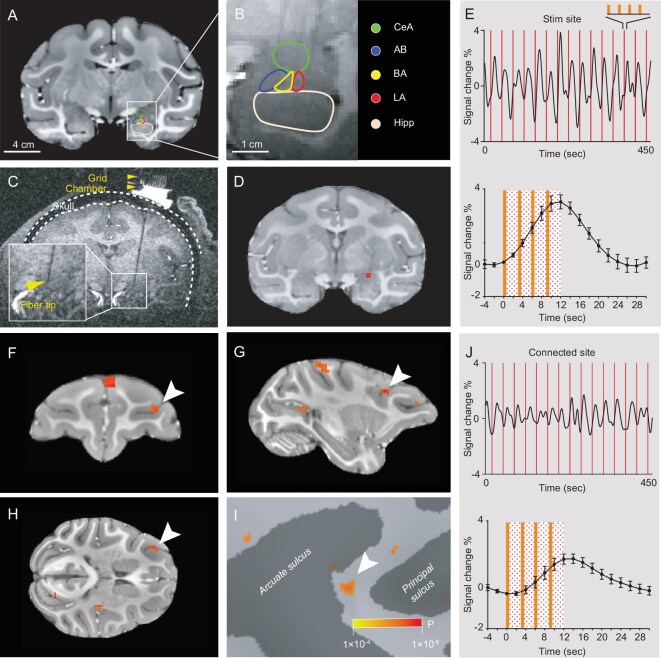
Identifying functionally connected sites in the brain following INS stimulation of single mesoscale sites in the amygdala. (A) A coronal section through the caudal amygdala. (B) Parcellation of the amygdala at the most caudal site shown in (A). CeA: central amygdala; AB: accessory basal amygdala; BA: basal amygdala; LA: lateral amygdala; Hipp: hippocampus. (C) Raw structural image indicating the optical fiber inserted through a grid in a chamber. (D) Activation at the laser tip in CeA (red voxel, intensity: 0.2 J/cm^2^, *P* < 1 × 10^–6^). (E) BOLD timecourse at the laser tip in D (TR = 2000 ms). Above: 15 consecutive trials; below: averaged timecourse (the dotted rectangle spans the duration of INS). Each red line: one trial of four pulse trains (see Methods). (F–H) Coronal, sagittal and horizontal view of a remote cluster activated in response to stimulation in (D) (*P* < 1 × 10^–4^). (I) Activation cluster (white arrow) in (F–H) shown on inflated brain surface. (J) BOLD timecourse at connected cluster (white arrow) in (f–h). Above: all 15 trials; below: averaged timecourse.

By inserting a 200 μm diameter optical fiber through a preinstalled grid into the amygdala (Fig. 2A–C), we stimulated discrete sites in the right amygdala of monkey K and determined the nuclear location of the stimulation site with a 0.3 mm precision (see Methods and previous study [32]). Periodic trains of pulsed infrared neural stimulation at the stimulated site (Fig. 2D) evoked BOLD signal response (Fig. 2E). As previously shown [31–33], functional connectivity at remote sites was evaluated by correlation to the stimulation site (see Methods). Fig. 2F–I present an example of a connected site in the frontal lobe with a timecourse with statistically significant correlation with the INS stimulation (Fig. 2J). A correlation value was obtained for every voxel in the brain; only voxels of high statistical significance (T-test *P* < 1 × 10^−3^, see Methods) were further studied. Reproducibility was evaluated using comparisons of half-runs (e.g. even vs odd runs, Fig. S2 and previous study [32]). Reliability of activation was also evaluated by examining different thresholds; generally, with larger *P* values (less stringent threshold), activation sizes increased, but activations remained in a similar location (Fig. S3 and previous study [33]; for an unthresholded cortical map, see Fig. S4), supporting the reliability of the activation location.

### Distribution of global cortical connections from CeA, BA and LA

**Figure 3.**
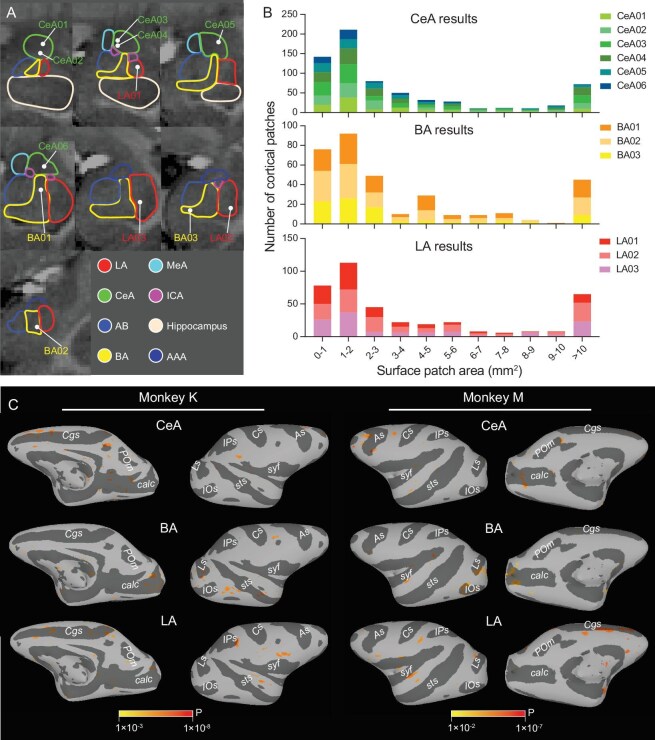
Mesoscale brainwide connections of the amygdala. (A) The white dots represent the INS stimulation sites in the right amygdala. CeA (green contour, six sites), BA (yellow contour, three sites), LA (red contour, three sites). LA: lateral amygdala; BA: basal amygdala; AB: accessory basal amygdala; CeA: central amygdala; MeA: medial amygdala; ICA: intercalated cell masses; AAA: anterior amygdala area. (B) The stacked histogram for patch size of brainwide cortical activations. The x axis represents the size of patches in millimeters squared. The y axis represents the number of patches of different sizes. Each color represents a stimulation site in monkey K, namely six sites in CeA (upper row, shades of green), three sites in BA (middle row, shades of yellow) and three sites in LA (lower row, shades of red). (C) Whole-brain activations evoked by single stimulation sites (one site for each of CeA, BA and LA) mapped on inflated hemisphere (ipsilateral to the stimulation) of monkey K and monkey M. Both medial view and lateral view are presented. Ps: principal sulcus; As: arcuate sulcus; Cs: central sulcus; IPs: intraparietal sulcus; syf: sylvian fissure; sts: superior temporal sulcus; Ls: lunate sulcus; IOs: inferior occipital sulcus; Cgs: cingulate sulcus; POs: parietal-occipital sulcus; POm: medial parieto-occipital sulcus; calc: calcarine.

To obtain a comparison of amygdala-stimulated networks, for each animal (monkey K, monkey M), we examined data acquired within a single animal (Fig. 3A shows an example shown of monkey K: six sites in CeA, three sites in BA and three sites in LA). Note that, unlike anatomical tracer injections, which tend to fill more of the amygdala (e.g. a subdivision), this study has sampled very focal (millimeter-sized) locations in different parts of the amygdala. Voxels with significant *P* values were selected for subsequent statistical analysis (see Methods and previous study [32]). As shown in Fig. 3B & C, activations from single site stimulation appeared patchy and mesoscale in size. The distribution had a sparse appearance and spanned multiple brain areas. This was seen consistently across every stimulation site in both monkey K and monkey M (examples shown in Fig. 3C). Patch sizes from stimulation in CeA, BA and LA were predominantly (64% of patches) less than 3 mm^2^ in size (Fig. 3B; for monkey M, see Fig. S1), although some activations that appear large (>10 mm^2^, <10%) at this threshold were, at higher threshold, composed of smaller patches. Note that, while the size of mesoscale domains is dependent on the threshold used, the locations of these activations remain stable and distinct (see Fig. S3). Thus, what we show in this study are the activations that have the strongest functional connections (highest correlations) with the stimulation site, providing a view of the ‘backbone’ of a functional network. Nodes within this functional network may be modulated in size and strength during natural behavior.

**Figure 4.**
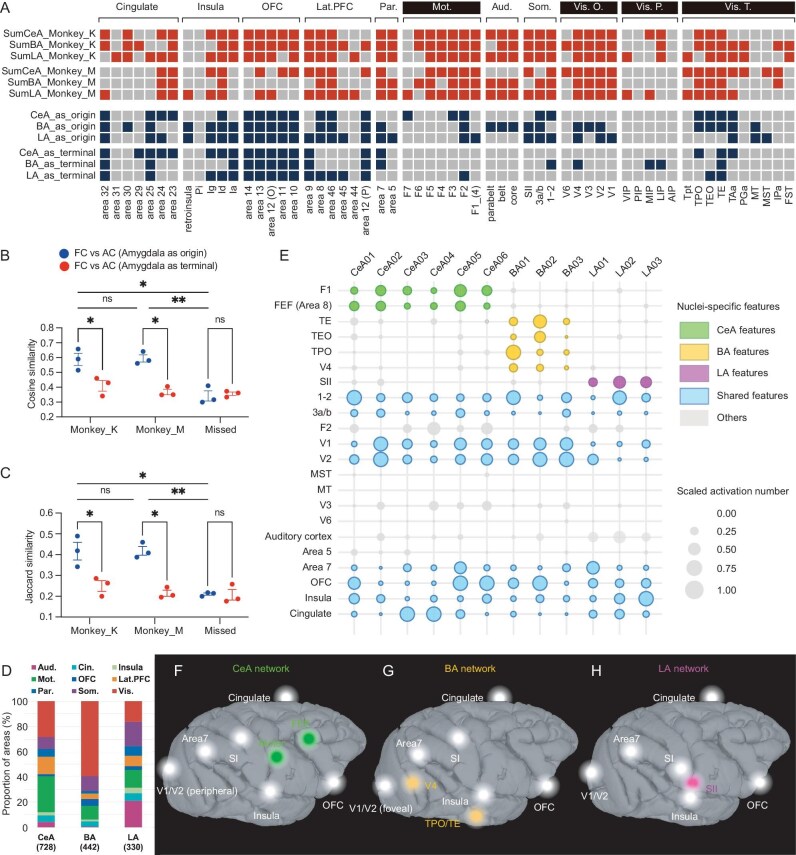
Cortical distributions of CeA, BA and LA networks. (A) A comparison of connectivity revealed by INS-fMRI and by anatomical tracers. Upper six rows: red represents the presence of functional connections in monkey K and monkey M. Lower six rows: blue represents the combined results of anatomical connections originating from the amygdala and anatomical connections to the amygdala originating from the cortex (based on http://cocomac.g-node.org, see Methods). T.: visual system (temporal); Vis. P.: visual system (parietal); Vis. O.: visual system (occipital); Som.: somatosensory cortex; Lat-PFC: lateral prefrontal cortex; Par.: parietal cortex; OFC: orbital frontal cortex; Mot.: motor cortex; Aud.: auditory cortex; Pi: parainsula; Ig: granular insula; Id: dysgranular insula; Ia: agranular insula. (B and C) Jaccard similarity and cosine similarity between functional connectivity (FC) and anatomical connectivity (AC); the red color represents similarity between FC and AC to the amygdala originating from the cortex, the blue color represents similarity between FC and AC that originated from the amygdala. Results are from two monkeys (monkey K and monkey M), and from stimulation sites out of the amygdala (missed) in monkey K. (D) Proportional composition of cortical connections from CeA, BA and LA in monkey K (e.g. for all stimulation sites in CeA, the # voxels in an area connected to CeA/total voxels connected to CeA). (E) Global distribution of activation evoked by different stimulation sites. Each column illustrates activation from a single site (six in CeA, three in BA, three in LA). The colors represent that the brain areas have outstanding and consistent activations from stimulating sites in CeA (green), BA (yellow) and LA (magenta). The blue color represents that all stimulation sites evoke activations in this brain area. The size of bubbles represents the number of voxels evoked by each stimulation site in each brain area. The numbers were scaled by each stimulation site. (F–H) Summarized global networks involving CeA (F), BA (G) and LA (H), respectively. The colored nodes represent areas dominated by CeA, BA or LA; the white nodes represent areas receiving similar prominence of connections from CeA, BA and LA.

We then examined the brainwide connectivity distributions and their similarity to published anatomical connectivity. Using D99 (version 1.2b) [37] parcellation, brain areas were classified largely by function into cingulate, insula, orbitofrontal (OFC), lateral prefrontal (Lat. PFC), parietal (Par.), motor (Mot.), auditory (Aud.), somatosensory (Som.), visual occipital (Vis. O.) and visual parietal (Vis. P.), and visual temporal (Vis. T.) (see listing of areas at the bottom of Fig. 4A). Although the stimulation sites sample only a small portion of the amygdala, overall, the distribution of functional connections (Fig. 4A, upper six rows in red, rows 1–3 for monkey K and rows 4–6 for monkey M) is largely in line with the known distribution of anatomical connections (Fig. 4A, lower six rows in blue). For example, we found stimulation areas that confirmed prediction based on the known major anatomical connections of the amygdala with the insular areas Ig, Id and Ia, orbitofrontal and prefrontal areas 11,11, 12, 13 and 14, and visual association areas TPO, TEO, TE and TAa in the upper bank of the sts. Surprisingly, the connectivity with motor and premotor areas was less supported based on anatomical studies, except for premotor area F2. However, the results were consistent across the subject monkeys M and K.

To systematically compare the congruence between functional connectivity and anatomical connectivity, we compared the amygdala functional connectivity matrices of two monkeys with anatomical matrices of two directions (axons to/from amygdala), calculating their cosine similarity (cosine of angle between two vectors, Fig. 4B) and Jaccard similarity (size of intersection divided by size of union, Fig. 4C). Additionally, we computed the functional connectivity matrices for stimulation sites outside the amygdala (missed) as a control. The results demonstrated that the amygdala functional connectivity matrices of both monkeys exhibited greater similarity with the anatomical matrices summarizing axonal connections originating from the amygdala, in contrast to those terminating in the amygdala. We note that this result is consistent with previous observations that stimulation-evoked connectivity is biased towards ‘anterograde’ directions [12,31,38]. Thus, the areal connectivity of INS stimulation is consistent with previous anatomical studies based in relatively large injection sites; however, the primary and new finding is the mesoscale patchiness of connectivity.

There were also substantial differences. The matrix reveals that functional connectivity is present in some areas where anatomical connections are not prominently observed, such as the motor cortex, and areas on visual pathways and visual cortex (Fig. 4A, marked by white text on a black background), potentially reflecting secondary connections. Another difference lies in the degree of ‘common connectivity’ that CeA, BA and LA share with single cortical areas. For example, all three subdivisions exhibit extensive bidirectional connections with the OFC. However, anatomical data reveal limited shared connectivity of CeA, BA and LA with motor cortex, somatosensory cortex and visual cortex (Fig. 4A). The differences in proportional connectivity reported in anatomical studies and this study are likely due to (i) the secondary connections revealed by INS and (ii) the small (mesoscale) size of the volume of stimulated projection neurons.

We also compared the relative distributions of connectivity between the three nuclei. Out of the total number evoked from all stimulation sites of each nucleus (CeA: 728, BA: 442 and LA: 330), the percentage of connections associated with each different brain area (grouped by function) exhibit distinct landscapes across nuclei (Fig. 4D). We noted a few distinct characteristics in the three distributions. Relative to BA and LA, CeA had the most functional connections with the motor cortex (28.4%) and the Lat. PFC (13.6%). Relative to CeA and LA, BA had the most with the visual cortex (59.6%). And, relative to CeA and BA, LA had the most with the auditory cortex (21.0%) and the somatosensory cortex (19.2%). By contrast, CeA, BA and LA exhibited similar proportions of functional connectivity with the cingulate cortex (∼5%).

To investigate the nature of the connections in finer detail, we evaluated the relative prominence of activations in different cortical areas from each of the stimulation sites in CeA, BA and LA (Fig. 4E). To compare the global distribution of connections across stimulation sites and to identify the unique or shared features of stimulation sites from different nuclei, we calculated the activation number (# voxels) in each brain area for each stimulation site. We scaled the numbers by stimulation sites using: scaled x = (x – min(x))/(max(x) – min(x)), where max(x) and min(x) are the maximum and minimum values, respectively, for each stimulation site. As seen in the top nine rows, some areas are dominated by connections from CeA (green: primary motor cortex [F1], frontal eye field [FEF]), BA (yellow: TE, TEO, TPO, V4) and LA (magenta: secondary somatosensory cortex [SII]). Other cortical areas connected with amygdala have significant contributions from all three nuclei (sensory areas 3a/b, 1–2, V1, V2, motor F2, and area 7, OFC, insula, cingulate, represented in blue). Yet other areas have relatively weak connections with amygdala (gray: V3, MT, MST, area 5, V6). These distinctions are summarized on brain views (Fig. 4F–H). Thus, the amygdala is extensively connected, directly or indirectly, with most cortical areas; from these sites of stimulation, it appears that dorsal and mediodorsal pathways are more weakly connected; this is consistent with anatomy for area 5 and V6, but not for MT, MST and V3. However, it is possible that we did not stimulate the sites connected to these areas.

### Similar but distinct functional connectivity in the cingulate cortex, insula and OFC

**Figure 5.**
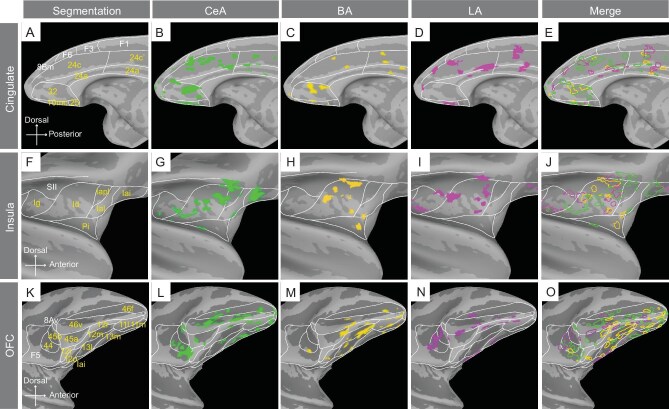
Functional connections with cingulate cortex, insula and OFC. Topography of connected areas in cingulate cortex (B–E), insula (G–J) and OFC (L-O). Segmentation of the brain areas is shown in the first column (A, F, K). Merged views of CeA, BA and LA are shown in the last column (E, J, O). Iai: intermediate agranular insula; Iapl: posterior lateral agranular insula; Ial: lateral agranular insula. The results are masked by cingulate, insula and OFC for the purpose of highlighting results in these areas.

Three of the major cortical connections of amygdala include the cingulate cortex, insula and the OFC [7]. Here, we illustrate the connections of these sites with the CeA, BA and LA nuclei of the amygdala (Fig. 5). The first general observation is that, within each of the activated cortical areas, connected sites appear patchy. For CeA stimulations (Fig. 5B,G,L), patchy activations were observed in four areas: (i) cingulate areas 23, 24 and 32, and medial orbitofrontal area 10; (ii) insular areas in the lateral sulcus (lg, ld, lapl), as well more infra-orbital insular areas lai and lal; (iii) lateral prefrontal areas 12, 44, 45 and 46, as well as (iv) infraorbital areas 11 and 13. The stimulation of BA sites (Fig. 5C,H,M) and LA sites (Fig. 5D,I,N) elicited similar activation profiles in the cingulate, insular and PFC (BA activations in cingulate and insula are consistent with previous research [32]). Some notable differences include: (i) in contrast to stimulation of CeA and BA, LA shows little activation in area 32 (see also Fig. 4A); and (ii) stimulation of BA shows little activation in anterior insular area Iai or in posterior Ig. However, closer inspection reveals that the respective activations of CeA, BA and LA are largely non-overlapping within each area (Fig. 5E,J,O, see merged). Overall, while this spatial comparison shows that CeA, BA and LA share common cortical targets, the mesoscale connectional architecture comprises largely distinct patchy territories within each cortical area, suggesting a degree of functional segregation.

### Mesoscale cortical connection patterns from each of CeA, BA and LA

**Figure 6.**
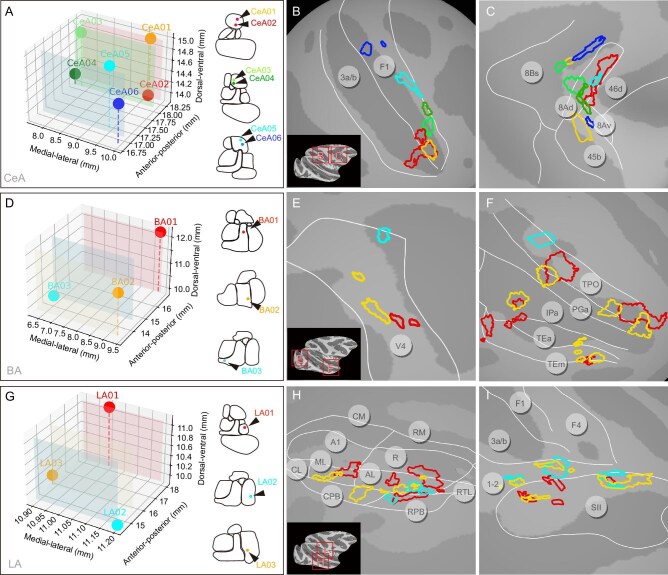
Local cortical topography of connections from single amygdala nuclei. Activations from different stimulation sites within each of CeA, BA and LA were mapped onto the cortical surface (*P* < 1 × 10^–3^). (A, D, G) Stimulation sites are shown in 3D coordinates (left) and in rostro-caudal contour cartoons (right). (A–C) Six stimulation sites in CeA revealed connected sites mostly in F1 (B) and FEF Area 8 (C). (D–F) Three stimulation sites in BA revealed connected sites in area V4 (E) and in ventral visual pathway TP, PG, IP, TE (F). (G–I) Three stimulation sites in LA revealed connected sites in auditory belt/parabelt areas AL, ML, CPB, RPB (H) and somatosensory areas 1–2 and SII (I). A1: primary auditory area; R: rostral area; CM: caudomedial belt region; AL: anterolateral belt region; ML: middle lateral belt region; RPB: rostral parabelt region; CPB: caudal parabelt region.

We next examined the connections from different stimulation sites within each of CeA, BA and LA to single cortical areas. These regions were selected based on the presence of activation, for a given cortical area, across multiple stimulation sites (as shown in Fig. 4E). As shown in Fig. 6A, the six stimulation sites in CeA all led to activations in the primary motor cortex (Fig. 6B) and area 8 of FEF (Fig. 6C); these showed distinct and largely non-overlapping distributions that appeared to be topographically organized. The patches corresponding to the four stimulation sites in posterior parts of CeA (CeA01, 02, 03 and 04) were mainly distributed in the lateral part of the primary motor cortex (Fig. 6B), potentially corresponding to the head and face motor areas [39,40]. By contrast, the connections corresponding to the two anterior CeA stimulation sites were located more medially in the primary motor cortex, possibly in the hand motor area. Interestingly, the activations in the primary motor cortex arising from the spatially closer stimulation points (CeA01 and CeA02, CeA03 and CeA04, CeA05 and CeA06, each less than 1 mm to each other) were also closer to each other on the cortical surface (Fig. 6B). For the FEF (Fig. 6C), most connection sites were located within area 8Ad and also exhibited little overlap; however, unlike the motor cortex, there appears to be some interdigitation of the activations from CeA anterior (CeA01 and CeA02) vs CeA posterior sites (CeA05 and CeA06). In a second monkey (monkey M), patchy activations were observed in both F1 and FEF (Fig. S5A–C).

The connections corresponding to the three stimulation sites in the BA (Fig. 6D) led to activation of several patches in V4 with the red patches most lateral (foveal), the cyan patch most medial, and the yellow patches intermediate (Fig. 6E), suggesting some foveal to parafoveal topography. In the temporal lobe, area TPO contains alternating red and yellow patches anteriorly, indicating an interleaved pattern of connectivity, as well as a single cyan most posterior patch (Fig. 6F). Similar, albeit smaller, red and yellow patches were observed in TE and IPa. In a second monkey, patchy activations were observed in V4 and TPO/IPa (Fig. S5D–E).

The connections from the three stimulation sites in LA (Fig. 6G) showed both overlapping and interleaved distributions in higher order sensory areas, including auditory belt and parabelt regions (Fig. 6H) and secondary somatosensory cortex (Fig. 6I). As shown in Fig. 6H, connections in the auditory belt cortex were largely distributed in patchy fashion in AL (where red, yellow and cyan could be viewed as interleaved), with a few additionally distributed in ML, CPB and RPB. For somatosensory cortex (Fig. 6I), the connections are mainly distributed along the border of areas 1–2 (yellow and cyan interleaved) and secondary somatosensory cortex, with some overlaps in the connections of the three stimulation sites. In a second monkey, patchy activations were observed in auditory and somatosensory (SII, 1–2) areas (Fig. S5G–I).

In sum, we observed that stimulation of different sites within each of CeA, BA and LA resulted in patchy activations in connected cortical areas. Patches were largely distinct and non-overlapping, and exhibited distinct types of topography (e.g. topographic, interleaved). Data from a second monkey supported the patchy nature of activations. These examples also illustrate that single nuclei within amygdala may have a topographic relationship with one cortical area (e.g. BA with V4) and an interleaved pattern with another (e.g. BA with TPO).

### Mesoscale connection patterns from CeA, BA and LA to single cortical areas

**Figure 7.**
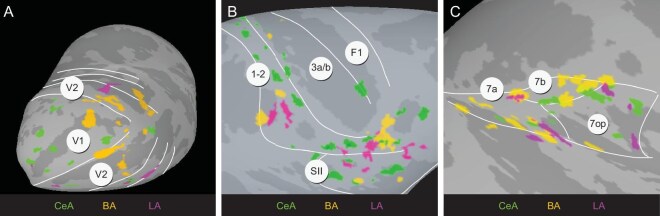
Connectivity patterns in cortical areas with activations from CeA, BA and LA. Topography of connected sites in V1/V2 (A), SI/SII (B) and area 7 (C), respectively.

We then examined how CeA, BA and LA connected to the same cortical area. As shown by the matrix of functional connectivity in Fig. 4E (blue bubbles) and Fig. 4F-H (white nodes), the brain areas that were strongly activated by all three nuclei include V1/V2 (Fig. 7A), SI/SII (Fig. 7B) and area 7 (Fig. 7C).

In V1 and V2 (Fig. 7A), BA's connections appeared heavily biased towards the foveal region (on the lateral convexity, yellow) and connections from CeA were predominantly localized in the peripheral regions (green), consistent with an association of BA with foveal visual attention-related behavioral circuits and CeA with physiological reaction to peripherally appearing stimuli (see Discussion). Connections from LA are fewer and distributed in a patchy pattern in V1, V2 and V3 (magenta). Notably, the connections from each nucleus are non-overlapping. In SI/SII (Fig. 7B), connections with LA were seen at the border between SI and SII and in the facial sensory area of area 1–2 (magenta), while the connections of BA (yellow) were distributed primarily in areas of area 1–2 corresponding to face and anterior parts of SII. Connections with CeA (green) were more broadly distributed across SI and SII. Patches were largely non-overlapping. For area 7 (Fig. 7C), the connections from CeA, BA and LA were also largely non-overlapping, with BA more dominant in 7a and 7b, LA in 7a and 7op, and CeA in 7b and 7op.

## DISCUSSION

Our study examines the cortical activations obtained by focal INS stimulation of sites in BA, LA and CeA nuclei of the macaque amygdala revealed by mapping in 7-T fMRI. The matrix and the details of the activation patterns can be summarized in four main points:

1. **Brainwide networks using INS-fMRI:** INS-fMRI reveals both primary and secondary functional connections, beyond the direct connections revealed by anatomical tracer studies, thereby providing a brainwide view of functional networks (Fig. 4A). In addition, matrix comparison of amygdala-cortical connections revealed by INS and known anatomical connectivity revealed similarities primarily with ‘amygdala as the origin’ (Fig. 4B and C), consistent with the ‘anterograde’ bias of the INS method [31].
2. **Amygdalo-cortical interaction mediated via functionally specific mesoscale activations:** Activations in cortical areas are consistently mesoscale (primarily one to a few millimeters) in size (Fig. 3B), raising the possibility of contacting specific types of mesoscale functional units in target cortical areas. Generally, the mesoscale patches (both those from the same stimulation site and from different sites in CeA, BA and LA) are non-overlapping (Figs 3–6, Fig. S5) and form topographic or interleaved arrangements (Figs 6 and 7). This is the first evidence that limbic inputs project to distinct functional units within target cortical areas.
3. **CeA-, BA- and LA-cortical networks comprise common cortical target areas and CeA-, BA- and LA-specific cortical areas.** The current sample of stimulation sites shows that CeA, BA and LA nuclei all project to a common set of cortical areas (Fig. 4F–H), and, in addition, target CeA-, BA- and LA-specific areas (CeA: F1, FEF; BA: V4, TE, T EO, TPO; LA: Aud, SII) (Fig. 4E, Fig. 7). Cortical areas with the strongest connectivity included the limbic areas the cingulate, insula and infraorbital cortex (Fig. 5), and the somatosensory cortex, visual cortex, and area 7 of the parietal lobe (Fig. 7). Interestingly, the spatial distribution of some cortical connectivity patterns revealed the presence of topographic specificity (somatosensory cortex SI and visual cortex V1) or interleaved distributions (area 7).
4. **Proposal basis vector for behaviors.** The key novelty is that, with our mapping technique, we are finding that distinct mesoscale sites in one brain area connect to distinct mesoscale sites in another area; the collection of multi-areal nodes activated from a single site in the amygdala comprises one behavioral vector. The excitement is that this network specificity suggests these sets of multi-areal mesoscale vectors embody our behavioral repertoire, and that our behavior may actually be quite quantifiable when reduced to such basis vectors.

### Methodological and statistical considerations

We evaluate the methods for identifying remote activations. Normally, causal fMRI studies use FDR-corrected *P* values based on generalized linear model (GLM) analysis to evaluate statistically significant BOLD response; from this, connectivity between stimulated and connected sites is inferred. BOLD response evoked at connected sites can be more robust with stronger stimulation paradigms (e.g. corrected FDR *P* values < 0.00005 [41]) and weaker with more focal or more cellularly specific stimulation (e.g. and *P* < 0.05 [42]). Here, INS leads to activation of a focal cluster of connected neurons and a relatively small BOLD signal (see Fig. 2). Statistics are well within published standards (FDR-corrected *P* < 0.05, range 1.9 × 10^−3^–1.4 × 10^−2^) and reveal consistent mesoscale connections between stimulated and connected sites.

The reliability of these activations is further supported by (i) half trial vs half trial reproducibility [32] (Fig. S2A&C), (ii) stability of activations across thresholds (Fig. S3), and (iii) alternating stimulation of two sites leads to two distinct sets of reproducible responses (Fig. S2B). Furthermore, similar-sized modularity is observed across brain areas, across stimulation sites, and across animals; this modularity shows complementary (non-overlapping) organization of connections (from CeA, BA and LA). Together, these point to the presence of non-random, non-artifactual, inherent structure in brain connectivity.

### Brainwide mapping using INS-fMRI

**Single functional nodes have brainwide influence:** We examined the spatial specificity and organization of brainwide networks revealed by INS and high-resolution fMRI (Fig. 7) and addressed the question of ‘What is the remote functional reach of a ‘single’ mesoscale node?’ Hu and Roe [12] showed that a local-scale stimulation of a single functional domain (column) in V2 elicits a pattern of intra-areal and inter-areal columns that repeats across different functional modalities in V2, demonstrating a canonical (∼10–12 columns) columnar microcircuit that serves distinct feature modalities. Here, we show the presence of analogous mesoscale brainwide circuits, a finding that reflects conceptually novel mesoscale-to-mesoscale specificity at global scale [32].

**Feedforward bias and circuit inferences:** Previous studies have shown that stimulation of a single cortical node leads to activation of middle layers at connected sites, consistent with that in anatomical feedforward connectivity [31,38]. Consistent with this, our method reveals stronger similarity with ‘anterograde’ than with ‘retrograde’ connection patterns (see matrix in Fig. 4A). For example, stimulation of amygdala produces robust V2 activation, but there is an absence of activation in amygdala following stimulation of V2 (data not shown: 23 sites in V2, 20 trials per site, no activated voxels amygdala). This suggests that the connections from CeA to V2 might be disynaptic, possibly through pulvinar or SC or another cortical area such as V1 [43,44].

### The mesoscale architecture suggests functional specificity

**Similar functional architecture in amygdalo-cortical connections**. The connection patterns revealed here are highly reminiscent of those observed in the visual system (see Introduction and Fig. 1). We find that the functional activations induced by INS stimulation are largely non-overlapping and have parallel and interleaved architectures, a result that can only be appreciated by comparing the distribution of multiple connectivity sources. These patchy connections bring into focus distinctions that may not be apparent from traditional anatomical tracer injection studies [45,46]. Thus, we infer that, similar to that in the visual system, the architectural features of amygdalo-cortical connection patterns reflect the presence of functionally specific emotion-related influence.

**Functional interpretation of amygdalo-cortical networks: social networks for facial and body communication.** While we do not know the mesoscale organization of many cortical areas, we make inferences of CeA-, BA- and LA-specific circuits based on published literature. Here, we provide one example. We have shown that CeA, a nucleus known to have a strong role in autonomic and homeostatic control, is strongly connected with primary motor cortex F1 and eye movement area FEF. Growing evidence indicates the role of the amygdala in facial recognition and in the valence and meaning of facial emotions [18,47,48]. Thus, CeA-mediated coupling of certain motor behaviors, such as eye movements with autonomic changes in response to alerting signals, could underlie the association of CeA with F1, FEF and peripheral visual cortex (Figs 4F, 6B&C, 7A) (e.g. facial expressions and other social signals through gestures, postures, etc.) (Fig. 6B), whereas the association of BA with foveal regions of visual cortex and face regions of areas 1–2 (Fig. 6E and F, A and B) may be associated with the ventral pathway evaluation of facial gestures. Likewise, the connections of the BA and LA that project in an interdigitating fashion to temporal areas, may impact distinct object-based (e.g. face patches) or sensory-based modalities in ventral visual pathway (TPO/TE/V4) and sensory cortex (auditory belt areas, somatosensory areas 1–2/SII), respectively (Fig. 6E, F, H). The critical role of these amygdalofugal projections for the functionality of the temporal cortical areas has been demonstrated by comparing the activation of multiple face-responsive visual areas in temporal cortical areas before and after excitotoxic lesions of the amygdala. In the absence of input from the amygdala, these cortical areas failed to respond to the stimuli that activated them reliably before the lesion [49].

These findings align with two features of our current understanding of cortical function: (i) Some cortical functions rely on spatial topography such as cortical columns in the visual cortex [50] or stripes distributed across the motor cortex [51]. There is also evidence that within the motor cortex the topography for motor action interdigitates with regions for combining action and physiological functions such as arousal and pain [40]. Neurons aggregated in the same cortical functional domain share a functional processing goal (e.g. color, shape, disparity, motion in visual cortex, action vs interoceptive nodes in motor cortex, object vs face patches in temporal cortex). Thus, the connections of the amygdala to different units may indicate the amygdala's processing of various features through different internal neuronal clusters. (ii) There is integration of multiple sensory and motor functions. Such integration might occur within the functional cortical areas, or in higher-order cortices, or in subcortical structures. Each stimulation site we targeted is connected to multiple cortical areas representing the different axes of behavior, indicating that the amygdala plays a significant bridging and integrative role in the emotion-cognitive-sensation-motor circuit.

### Mesoscale organization of brain function and brain disease

One of the impacts of this study is revealing how limbic influence interfaces with different axes of sensory, motor, cognitive, limbic and autonomic function represented by various cortical areas. This can lead to hypotheses for clinicians to dissect various profiles of neurological or neuropsychiatric dysfunction. Given sufficient data, one could envision developing selective and targeted modulation of specific mesoscale nodes, e.g. deep brain stimulation for motor disease such as Parkinson's and neuropsychiatric disease such as depression and obsessive-compulsive disorder (see [52]). A possible advantage of mesoscale stimulation is the avoidance of unwanted side effects resulting from broader electrical stimulation in deep brain stimulation [25]. Beyond clinical impact, this view will also impact how engineers design brain interface technologies [53,54] and how computational neuroscientists model brain circuits in behavior and disease [55].

### Comparison with previous studies

To understand the connectivity patterns of the amygdala, the earliest and most direct method employed neural tracers to study connections from an anatomical perspective. This approach led to the recognition of the prominent connections between the amygdala and OFC, insula, and anterior cingulate cortex, and many other cortical and subcortical areas, establishing the structural basis for the affective modulation of multiple functions. Building on this foundation, studies employing electrical stimulation and neurochemical modulation have provided further understanding of effective connectivity of the amygdala; these revealed a broader set of functional connections, including those with the posterior cingulate, retrosplenial cortex, parietal cortex and temporal cortex [56,57]. At the whole-brain level, neurochemical modulation of the amygdala via designer receptors exclusively activated by designer drugs (DREADDs) has shown significant impacts on global brain networks [56,58].

The establishment of various standardized, large-scale human MRI datasets (some with multimodal data) enables populational analysis of human amygdala [59–61]. The variations in connectivity patterns for the same nuclei can reflect discrepant functional properties, which are often attributed to differing sampling strategies of the amygdala, indicating that connectivity can vary across different locations within the same subnucleus. Clinical research conducted on stereotactic electro-encephalography in epilepsy patients, through direct electrical stimulation, has observed connected areas shared by BA and LA, including OFC, insula, anterior cingulate cortex, and post-central gyrus, revealing temporal and spatial differences in the connectivity patterns of different nuclei [57]. Another study [62] applied electrical stimulation in awake epilepsy patients and evaluated the patients' sensations, revealing the integrative role of various nuclei in mediating emotional reactions and sensory functions, including visual, auditory and vestibular sensations. These studies indicated that the modulation of the amygdala affects not only areas directly connected to it but also the activity of secondary regions. Similar to existing anatomical findings, we observed connections between the amygdala and the OFC, insula and cingulate gyrus; in addition, focal connections with multiple areas, including the somatosensory, auditory, visual and motor cortices, exhibited distinct topographic mesoscale organizations. These topographies appeared to fall broadly into three classes described as parallel, interdigitating and convergent. Our study thus echoes and extends previous findings, revealing the fine-scale organization of how different axes of amygdala function (BA, LA, CeA) influence individual cortical areas as well as selectively integrate brainwide circuits for emotion-guided social behavior.

## MATERIALS AND METHODS

The methods used here for macaque monkey animal procedures, amygdala INS stimulation, data acquisition and analysis are similar to those described in [32].

### Macaque monkeys

Two hemispheres in two Rhesus macaques (Macaca mulatta) were used (monkey K: right amygdala, monkey M: left amygdala). We have analyzed and present here 12 stimulation sites from 12 sessions in monkey K (see Fig. 3A), and six stimulation sites from six sessions in monkey M (see Fig. S1, Fig. S5).

### Animal preparation and surgery

All procedures were in accordance with the National Institute of Health's Guide for the Care and Use of Laboratory Animals and with the approval of Zhejiang University Institutional Animal Care Committee. In an initial session, high resolution structural and vascular scans were obtained. Sites to be targeted in the amygdala were then planned and a grid was implanted over one hemisphere to aid in the systematic targeting of multiple sites in different nuclei of the amygdala. The animals were sedated with ketamine hydrochloride (10 mg/kg)/atropine (0.03 mg/kg) and anesthetized with 1%–2% isoflurane; then the animals were intubated, placed in a custom MR-compatible stereotaxic apparatus, and artificially ventilated. After local infiltration of skin with lidocaine 1%, a small incision was made in the scalp and a 0.5 mm small burr hole craniotomy was then performed at one of the grid site locations determined by previous structural scans for targeting CeA, BA and LA. During the entire procedure, the animal's body temperature was maintained at 37.5–38.5°C with a water blanket. Vital signs (heart rate, SpO2, end-tidal CO_2_, and respiration rate) were continuously monitored. During the scan, monkeys were maintained with sufentanil (2 to 4 μg/kg per hour continuous rate infusion; induction, 3 μg/kg supplemented with 0.2%–0.5% isoflurane). Vital signs (heart rate, SpO2, end-tidal CO_2_, respiration rate, temperature) were continuously monitored.

Single optical fibers were inserted into the brain according to the designed stimulation sites. Each fiber typically targeted 3–4 sites in a single penetration and data were collected from stimulation at each site. The fiber was then retracted from the brain, and a new fiber was inserted in a different planned penetration. Following data collection, the 0.5 mm burr hole in skull was then sealed by bone wax; no fiber was left in the brain. Single sessions were conducted once every 1–3 weeks. For terminal experiments in monkey K (which lasted 2–3 days), following the completion of data collection, the animal was given an overdose of euthanasia agent iv.

### INS setup and stimulation paradigm

To reach the amygdala in macaque monkeys, we used 6-cm-long fibers attached to a 2.5 mm diameter MR-compatibility ferrule. We used 200 µm diameter low-OH silica core fibers (0.22 numerical aperture) with 10 µm thick cladding of fluorine-doped silica, resulting in a total diameter (outer diameter) of 220 µm. Prior to the experiment, the optical fibers were inspected, cleaned and sterilized. Using a simple inverted microscope and appropriate optical mounting hardware, both the ferrule end and the free end of the fiber probes (the latter directly contacts the brain tissue) were inspected. To remove any particles from the core and cladding at the free end, the fiber may be drawn multiple times through a folded lens tissue wetted with isopropyl alcohol. Both ends must be free of contaminants such as dust or oil to achieve optimum transmission of the laser light.

To determine the position of the tip within the amygdala during the experiments, we conducted a structural scan prior to every INS stimulation run, which revealed a dark spot of signal dropout distinct from surrounding tissues (see Fig. 2C). Stimulation sites were further confirmed by the location of fiber tip BOLD activation. We applied INS paradigms previously shown to be effective at neuronal activation. We used a laser generator (multimode laser, Changchun New Industries Optoelectronics Tech. Co., Ltd. (CNI), Jilin, China, PG4000A) to generate pulsed infrared stimulation (1875 nm wavelength). As in our previous studies using INS stimulation [31–33], each trial consisted of four pulse trains (12 sec) followed by 18 sec to allow the BOLD signal to return to baseline. Each pulse train lasted 0.5 sec (100 pulses, pulse width 250 µs, delivered at 200 Hz), with 2.5 sec between each one of the pulse trains. This quadruple of pulse trains was delivered once every 30 sec and repeated 15 times (15 trials) for each run, 1 intensity per run (total period of 450 sec). Radiant exposures that were previously shown to be non-damaging [24,25] ranged from 0.1–0.5 J/cm^2^. To ensure data consistency and comparability in this study, only trials stimulated with 0.2 J/cm² intensity were included. The stimulation intensity was consistent during each run.

### Data acquisition procedure

Functional images of voxel size 1.5-mm-isotropic were acquired in a 7-T Magnetom MR scanner (Siemens, Erlangen, Germany) with a customized six-channel receive coil (inner diameter 6–7 cm) with a single-loop transmit coil (inner diameter 18 cm) and a single-shot echo-planar imaging (EPI) sequence (TE 25 ms; TR 2000 ms; matrix size 86 × 72; flip angle 90°). This coil provided improved homogeneity of temporal signal-to-noise ratio (tSNR) over regular surface coils, resulting in images with similar tSNR values (mean tSNR of gray matter ∼75). Functional images from the opposite phase-encoding direction were also acquired for correction of image distortion [63]. In addition, Magnetization Prepared Rapid Acquisition Gradient-Echo (MPRAGE) sequence was used to obtain structural images of voxel sizes 0.3 mm (monkey K) or 0.5 mm (monkey M) isotropic.

### Detection of significant responses

Structural and functional images in raw DICOM files from a Siemens scanner were converted to NIfTI [64] and AFNI (Analysis of Functional NeuroImages) format [65]. Functional images were preprocessed with correction for slice timing, motion, image distortion and baseline shift. Significant responses were identified in a commonly used GLM approach, in which the timecourse of each voxel was regressed on the stimulus predictor (see Fig. 2). The stimulus predictor was the convolution between laser onsets and the standard hemodynamic response function. Regression coefficients were subjected to T-tests. The BOLD signals presented in Fig. 2E and J were bandpassed at 0.01–0.08 using 3dBandpass. Only voxels with significant T-test *P* values were highlighted on top of the structural images (*P* < 1 × 10^−3^); the median FDR-corrected (Benjamini–Hochberg) *P* was 6.1 × 10^−3^ [range 1.9 × 10^−3^–1.4 × 10^−2^]. Individual voxel timecourses were extracted from EPI data with AFNI 3dmaskdump. Timecourses of percentage signal change were calculated at each timepoint tn as: [Signal(tn)-Signal(t0)]/Signal(t0). Timecourses were then averaged over repetitions (15 trials) and plotted. Each baseline was estimated with the mean MR signal over full timecourse. The analyses were done with software AFNI (version 21.0.20) [65], FreeSurfer (v6.0.0) [66], Nipype [67], Bash, R (4.0.2) and Python (3.11.6). Out of all the voxels in the brain, only those voxels with a statistically significant correlation with the stimulation site were considered. Significant voxels were visualized on skull-stripped structural images using the FreeSurfer v6.0.0 software package (https://surfer.nmr.mgh.harvard.edu/).

### Tests for reliability

To examine whether these significant sites represent reliable functional connections, several analyses were conducted to support the reliability of the activations. (i) Half and half analysis: To examine which voxels were reliable, runs were divided into two groups (i.e. even and odd runs) and GLM correlation analyses (as described above) were conducted. Similarity of the activation pattern supported the reliability of response (see Fig. S2). (ii) Alternative stimulation paradigm: To assess the resolution capability of INS for differentiating connected sites in response to varied stimulation sites, we inserted two optical fibers in the amygdala of monkey M, alternately stimulating sites within BA and LA, and conducted GLM analyses on BA/LA trials separately. The results indicated that trials involving stimulation of BA specifically activated TPO, while those stimulating LA specifically activated the auditory cortex (see Fig. S2B), mirroring findings from continuous stimulation of either BA or LA sites with a single optical fiber. This reflects INS's accuracy for spatial investigation of whole-brain networks. (iii) Stability across thresholds: Activation maps were examined using different *P* values (resulting in larger activation sizes with less significant *P* values). The corresponding activation patterns remain generally stable, reinforcing the reliability of the method functional connectivity between the amygdala and voxels with significant correlation (see Fig. S3 [33]). (iv) Similarity across animals: We compared, across animals, activation patterns following stimulation of the same (or very similar) sites in the amygdala (see Fig. 6 and Fig. S5 [32]).

### Image alignment

All structural and functional images were co-registered to the digital version of rhesus monkey atlas with AFNI command 3dAllineate and 3dNwarpApply. We used D99 digital atlas (version 1.2b) [37] for cortical segmentation, and SARM digital atlas [68] for subcortical segmentation. The alignment was then manually examined according to an MR-histology atlas [69], as well as www.brainmaps.org for subcortical and brainstem sites; annotations of brain regions were then assigned to all voxels in the brain. Stimulation sites were determined in structural images on which the tip of the optic fiber was dark and distinct from tissues (see Fig. 2C) and in functional images based on functional activation (see Fig. 2D).

### Determining voxel number and cortical patch size

We counted the number of voxels in the whole brain (including cortex, subcortical and brainstem areas), determined using a brain mask (automated, then manually reconfirmed), and then determined by AFNI command 3dBrickStat. The number of voxels activated from each stimulation site, at specific thresholds (*P* < 1 × 10^−3^), were then determined and the percentage out of the total voxels calculated. For area-specific voxel counting, we applied the aligned atlas to acquire the certain number of activated voxels in different areas. For calculation of cortical patches, the voxels above the thereshold were first transformed using FreeSurfer command mri_vol2surf and mri_cor2label, and then measured using mris_anatomical_stats.

### Comparing with anatomical connectivity matrix

To obtain anatomical evidence of connections between the amygdala and various brain regions, we utilized the CoCoMac database (http://cocomac.g-node.org) to identify axonal projections originating from or terminating in CeA, BA and LA of the amygdala. Initially, we retrieved comprehensive lists of synonymous text IDs for CeA (62), BA (120) and LA (61). Our inclusion criteria were restricted to sites located within single nuclei, while we excluded sites encompassing multiple nuclei (for example ‘basolateral’ sites were excluded because they involve both BA and LA). Next, we generated lists of axonal projections by setting amygdala sites as the axon origin sites and axon terminal sites, respectively. Finally, we filtered projections that partially or completely overlapped with the targeted area, supporting the existence of such anatomical connections. For comparison with functional connections (Fig. 4A–C), these anatomical connections were manually attributed to corresponding brain regions of the D99 atlas. To compare the similarity between functional connectivity and anatomical connectivity, cosine similarity and Jaccard similarity were calculated between FC (pooled for CeA, BA and LA, respectively) and AC matrix. For control analysis refer to Fig. 3B and C. The steps and algorithms we used for control analysis are the same as the within-amygdala analysis. The only difference is that the stimulation sites for control analysis are not within the amygdala.

### Data visualization

Prism version 8.4.3 for mac, GraphPad software (La Jolla, CA, USA), was used for statistical analysis. Python version 3.11.5, package ‘matplotlib’ [70] and R version 4.0.2, package ‘ggplot2’, ‘ComplexHeatmap’, were used for data visualization.

## Supplementary Material

nwae473_Supplemental_File

## Data Availability

The data to evaluate the conclusions of this study are available within the article and the supplementary materials. Additional data are available upon request.
