# Supplementary material for: Brainwide mesoscale functional networks revealed by focal infrared neural stimulation of the amygdala
Source: Natl Sci Rev. 2024 Dec 24;12(4):nwae473. doi: 10.1093/nsr/nwae473 (PMC11960096; doi:10.1093/nsr/nwae473)
Supplement: nwae473_Supplemental_File [file nwae473_supplemental_file.docx]

**
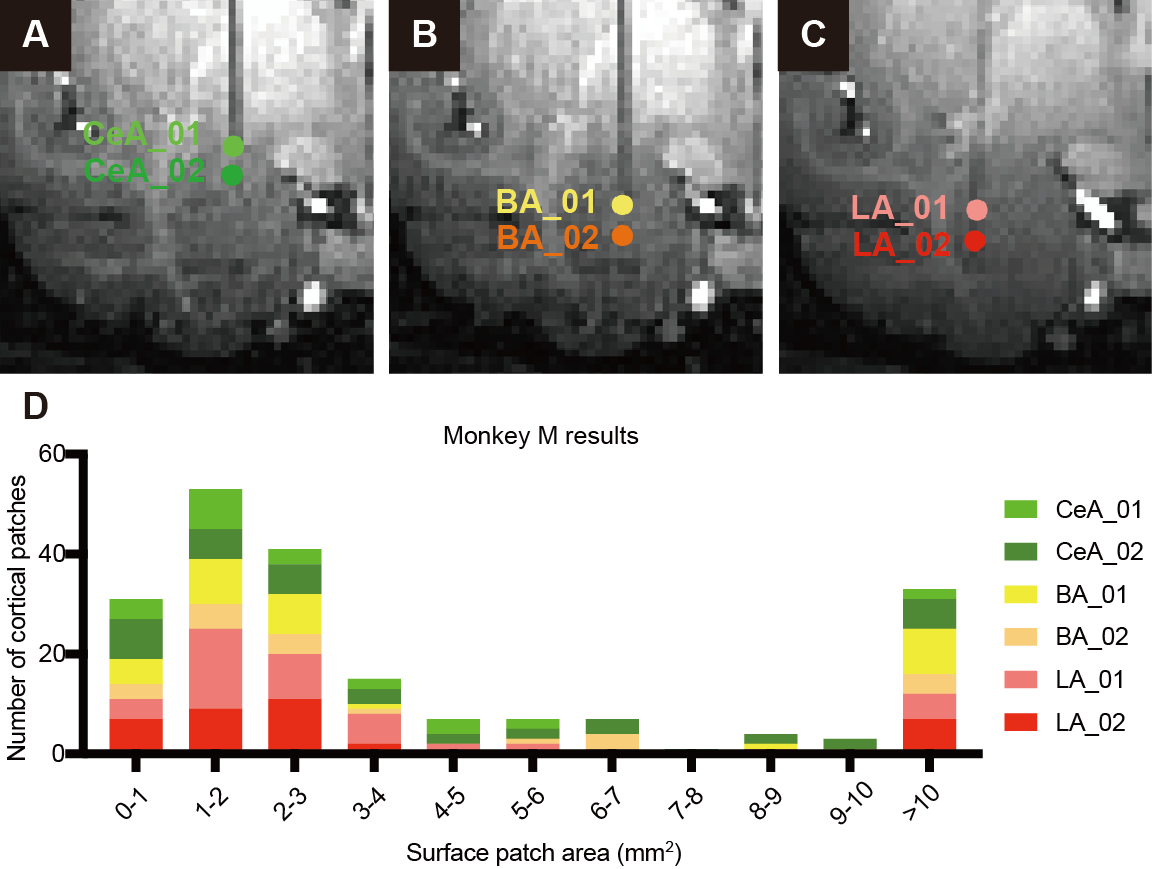
**

**Fig. S1.The stacked histogram for patch size of brainwide cortical activations (Monkey M).** The x axis represents the size of patches in millimeter square. The y axis represents the number of patches of different sizes. Each color represents the statistics of a stimulation site in monkey M, namely 2 sites in CeA (upper row, shades of green), 2 sites in BA (middle row, shades of yellow) and 2 sites in LA (lower row, shades of red).

**
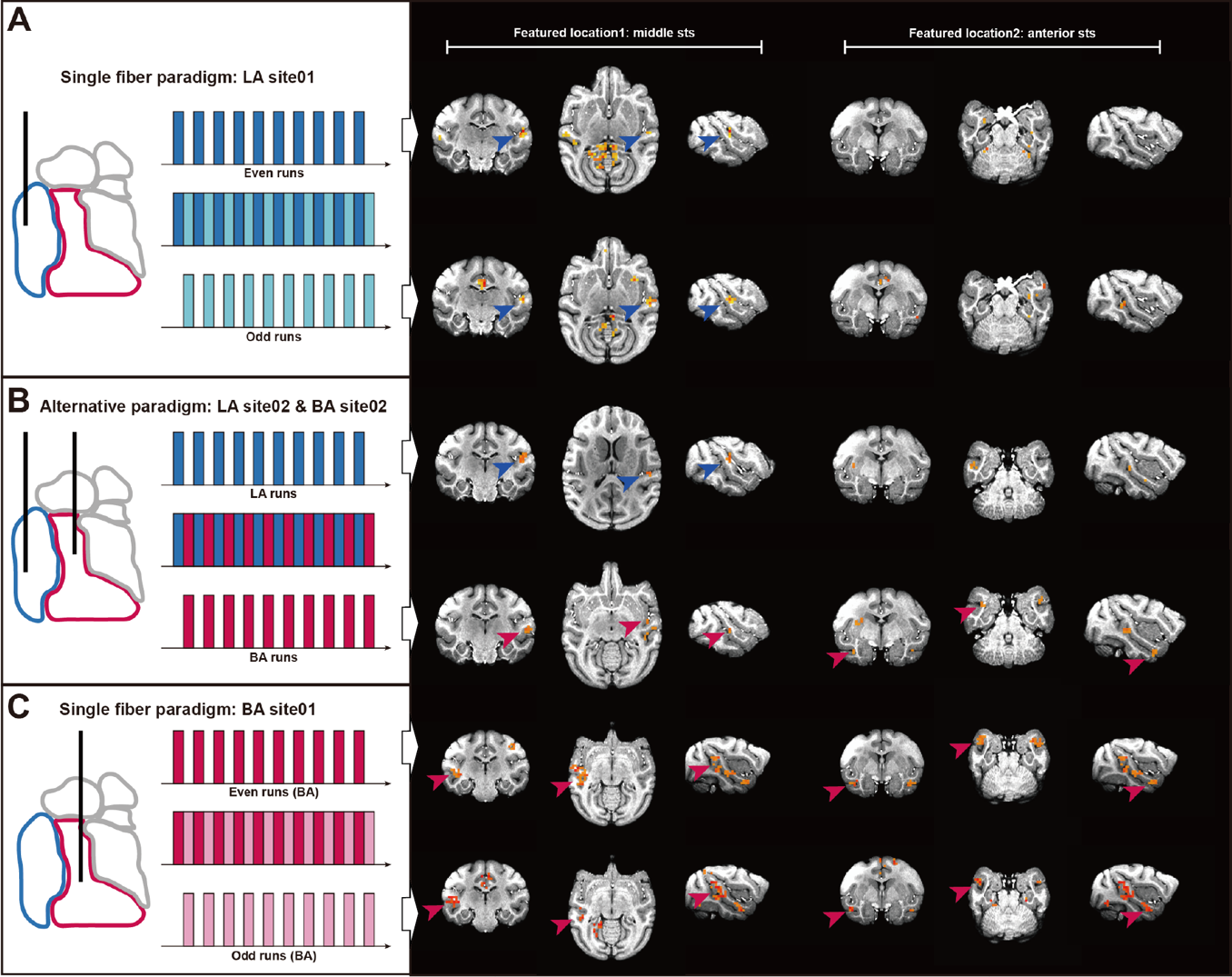
**

**Fig. S2. Half and half analysis and alternative stimulation paradigm.** We examined the reliability of brainwide activations to amygdala INS stimulation by comparing half trials. Left: half-half analysis (A&C) and alternating stimulation (B) were used. Right: Activations in two regions of the brain are shown (left: middle sts, right: anterior sts). (A) For an example stimulation site in LA, 20 trials were divided into even runs and odd runs and then analyzed using GLM model separately. (B) For a pair of stimulation sites, one in LA and another in BA, the stimulation of each was performed alternatively, each for 10 trials. (C) Same as in (A), except the stimulation site was in BA. The same threshold level was selected for all tests (p<5×10-3). Data from monkey M. The results in (B) indicated that trials involving stimulation of BA specifically activated TPO, while those stimulating LA specifically activated the auditory cortex, mirroring findings from continuous stimulation of either BA (A) or LA (C) sites with a single optical fiber.

**
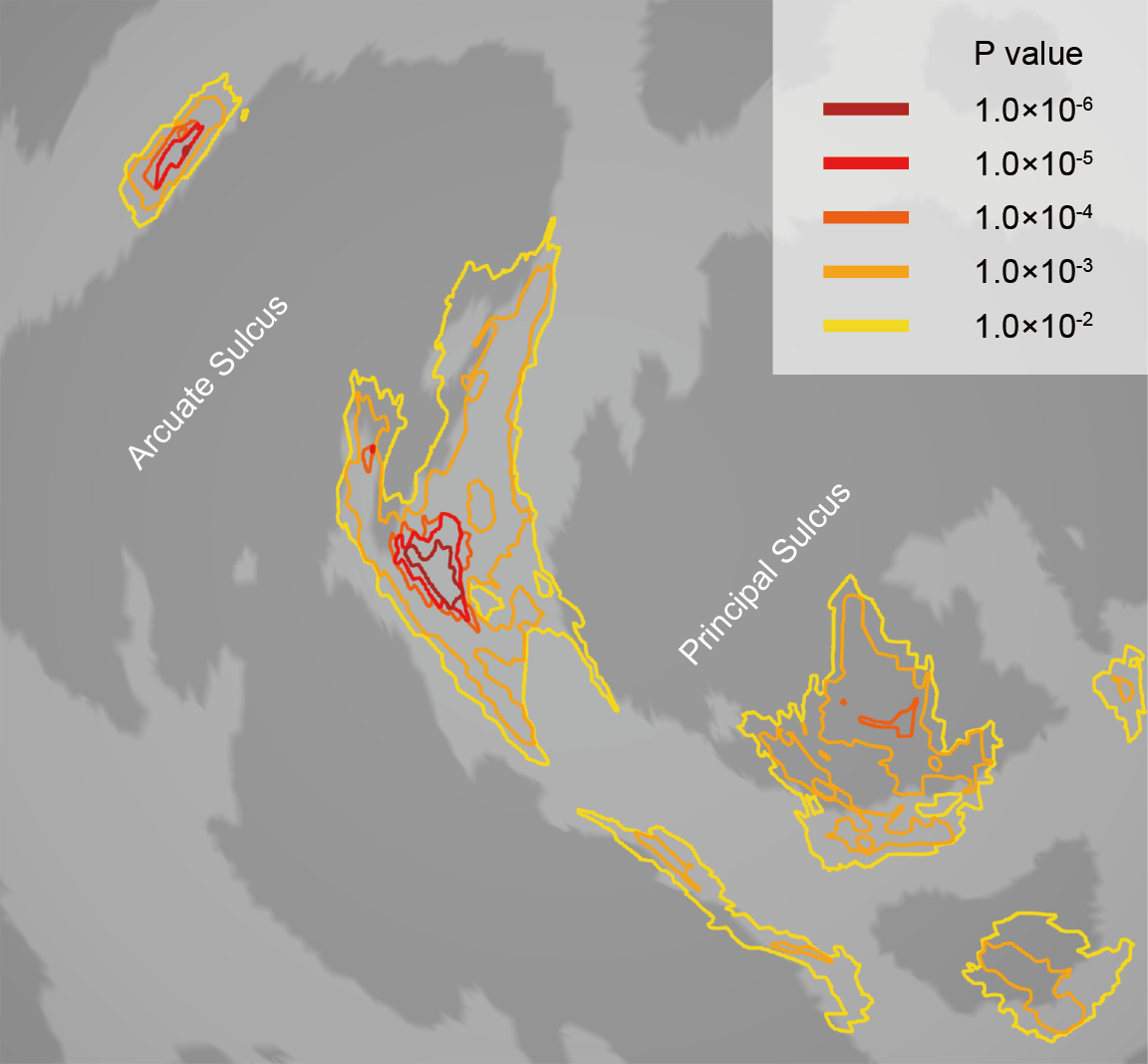
**

**Fig. S3. An example of the response patterns at different thresholding p values.** The activation evoked by stimulating at a site in medial CeA. The colors of the contours stand for the thresholding percentage. The relationship of thresholds and the corresponding p values are presented at the right upper corner. The main point is that, while the sizes of activations increase with lower threshold, the locations of activations remain largely stable. Our data emphasize the most significant activations seen (highest correlation values), reflecting the ‘backbone’ of the functional network.


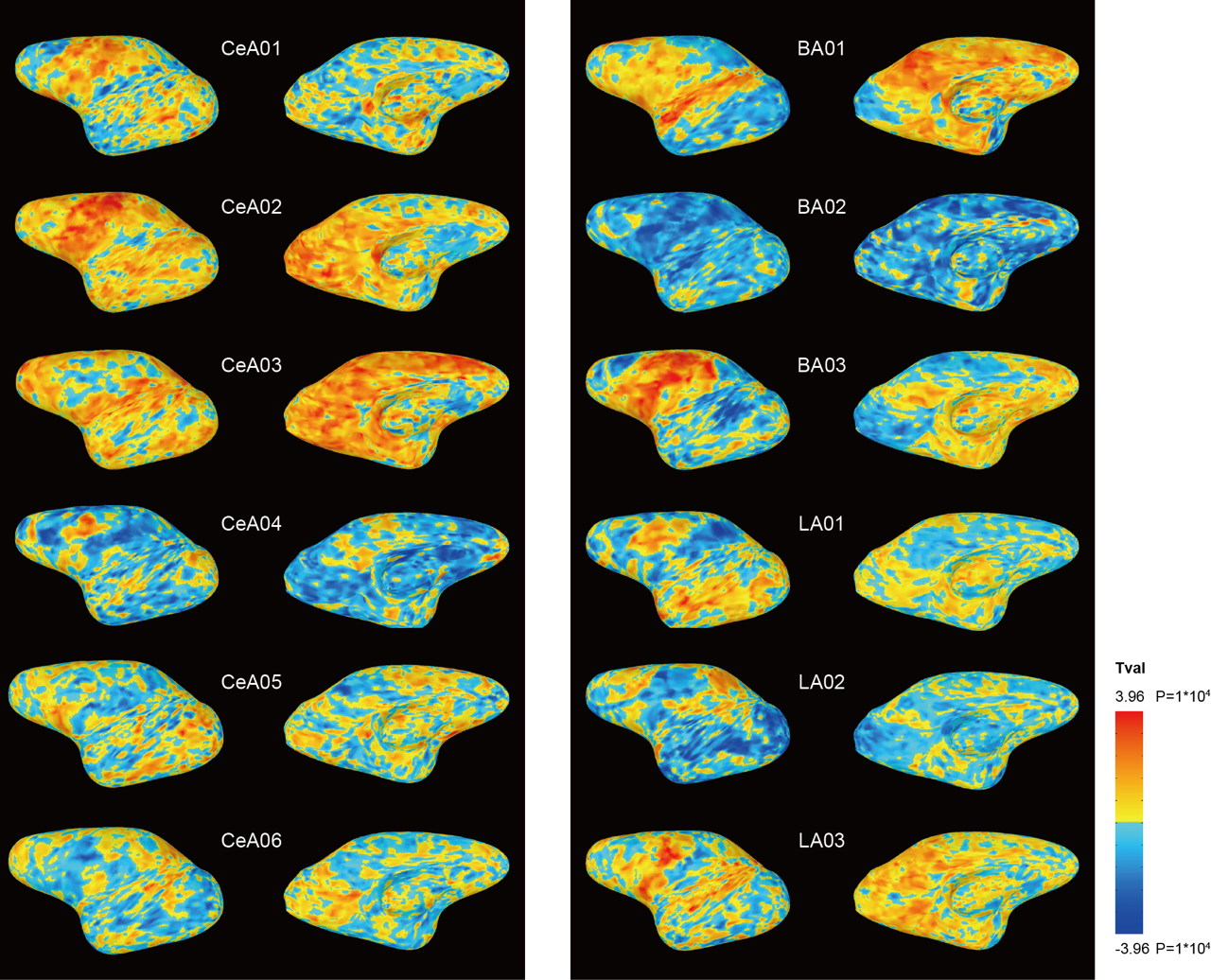


**Fig. S4. Un-thresholded cortical map of brainwide activation from stimulation sites in CeA, BA and LA (Monkey K).**

**
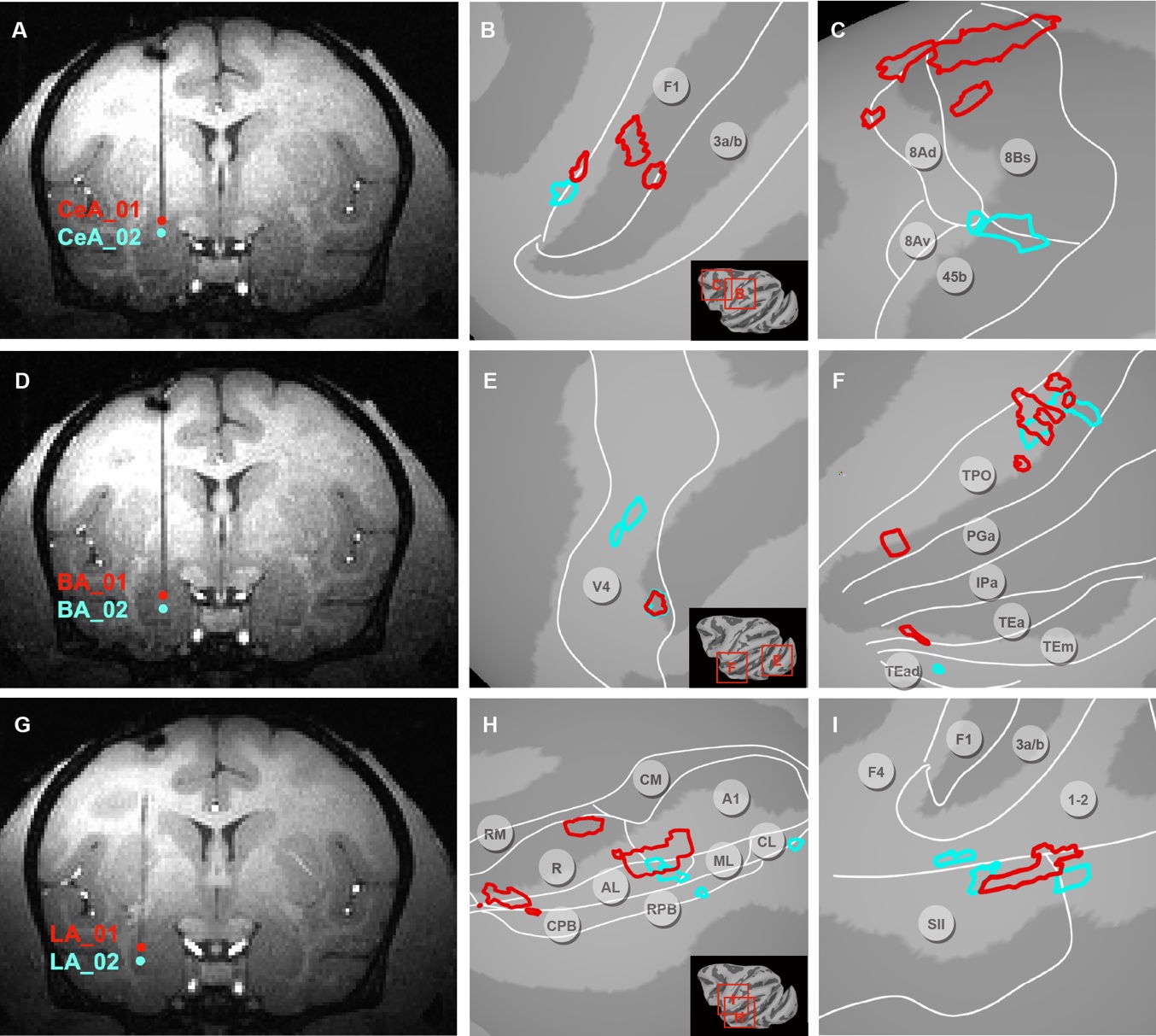
**

**Fig. S5. Local cortical topography of connections from the amygdala (Monkey M).** (A-C) Two stimulation sites (A) in CeA revealed connected sites in F1 (B) and FEF (C). (D-F) Two stimulation sites (D) in BA revealed connected sites in area V4 (E) and in ventral visual pathway TP, IP (F). (G-I) Two stimulation sites (G) in LA revealed connected sites in auditory areas (H) and somatosensory areas SII (I). Note that the colored patches (P<1×10-3) indicate activation locations and do not contain correlation strength information. Monkey M dataset: right brain.
